# Hepatic encephalopathy due to non-cirrhotic portal hypertension associated with non-alcoholic steatohepatitis and chronic hepatitis B: a Case Report

**DOI:** 10.3389/fmed.2025.1576827

**Published:** 2025-11-12

**Authors:** Huan Li, Jinhua Zhang, Wen Liu, Youwei Liu, Xiangming Yu, Shuyan Sun

**Affiliations:** 1 Department of Neurology, The 970th Hospital of PLA Joint Logistic Support Force, Yantai, China; 2 Department of Pathology, The 960th Hospital of PLA Joint Logistic Support Force, Jinan, China

**Keywords:** non-alcoholic steatohepatitis, hepatitis B virus, non-cirrhotic portal hypertension, hepatic encephalopathy, case report, liver biopsy, diagnostic challenges, therapeutic management

## Abstract

This case report examines the diagnostic and therapeutic complexities presented by a patient with hepatic encephalopathy resulting from overlapping pathologies of non-alcoholic steatohepatitis (NASH), hepatitis B virus (HBV) infection, and non-cirrhotic portal hypertension (NCPH). Highlighting the intricate relationship among these conditions, this study delineates the distinct and overlapping clinical features, diagnostic challenges, and therapeutic approaches. The patient exhibited atypical symptoms typical of NASH but lacked clear signs of cirrhosis, complicating both the diagnostic process and the therapeutic management. The diagnostic journey involved a nuanced assessment using multimodal imaging techniques, which were crucial in distinguishing between hepatic encephalopathy caused by cirrhosis and that due to NCPH. Treatment strategies had to be carefully tailored to address the specific etiological factors and pathology of the conditions involved, with particular attention to managing metabolic disorders such as insulin resistance and abnormalities in lipid and glucose metabolism, frequently observed in both NASH and HBV. The case underscores the need for a comprehensive and individualized approach in managing complex hepatic conditions, especially when conventional diagnostic criteria and treatment protocols face limitations.

## Introduction

Hepatic encephalopathy (HE), a complex neuropsychiatric disorder, has traditionally been associated with parenchymal liver diseases such as acute liver failure and cirrhosis; however, in recent years, increasing attention has been directed toward HE arising in the context of non-cirrhotic portal hypertension (NCPH), a condition characterized by elevated portal or visceral circulatory pressures in the absence of significant hepatic parenchymal injury. The diagnosis of NCPH is established through the demonstration of clinical features of portal hypertension (including splenomegaly, varices, or portosystemic collaterals), radiological or hemodynamic evidence of increased portal pressure, and confirmation of preserved hepatic architecture by liver biopsy, while excluding cirrhosis and advanced parenchymal disease by means of imaging, biochemical tests, and histological evaluation; depending on etiology, vascular imaging may reveal either obstruction, as in extrahepatic portal vein obstruction (EHPVO), or patency with subtle sinusoidal changes, as in idiopathic non-cirrhotic portal hypertension (INCPH) and porto-sinusoidal vascular disease (PSVD). Within this diagnostic framework, studies have reported the prevalence of minimal hepatic encephalopathy (MHE) in NCPH to range from 12% to 60%, varying by underlying pathology and diagnostic tools used; notably, in patients with EHPVO, nearly one-third develop MHE, with significantly elevated venous ammonia levels and more frequent spontaneous shunting in affected individuals, underscoring the central role of ammonia dysmetabolism and portosystemic shunts in the pathogenesis of HE in NCPH (1).

Viral hepatitis, particularly hepatitis B virus (HBV), constitutes a significant global health threat. The World Health Organization (WHO) estimates that viral hepatitis was responsible for approximately 1.3 million deaths in 2022, with nearly 1.1 million attributed to hepatitis B. Despite progress in controlling viral hepatitis, global testing and treatment coverage remains suboptimal. In high-burden regions, such as Africa and Southeast Asia, the diagnosis and treatment rates for hepatitis B and C remain extremely low, exacerbating the risk of disease transmission and contributing to disease progression, potentially leading to complications, including NCPH (2).

Non-alcoholic steatohepatitis (NASH), a severe stage of non-alcoholic fatty liver disease (NAFLD), has shown a global increase in prevalence in recent years, emerging as a significant public health burden. NASH is characterized by hepatocellular steatosis, inflammation, and hepatocellular injury, which disrupts normal liver architecture and function, thereby increasing the risk of portal hypertension. When NASH coexists with viral hepatitis, hepatic pathology becomes increasingly complex and severe. The interaction between these two etiologies may accelerate hepatic fibrosis, leading to progressive hepatic vascular damage, which can trigger or worsen non-cirrhotic portal hypertension (NCPH) and heighten susceptibility to severe complications such as hepatic encephalopathy (HE) (3).

Existing research on NCPH-associated HE has primarily focused on cases with a single etiology; however, clinical insights into cases involving NASH in combination with HBV remain limited. Such cases are often more complex, posing significant challenges in diagnosis and management. In light of this, we present a case of NCPH-associated HE in the context of NASH and HBV to explore its clinical characteristics, diagnostic challenges, disease associations, imaging findings, and treatment approaches. This study aims to provide clinicians with a valuable reference for managing such complex cases and to enhance overall understanding of this disease, its diagnosis, and treatment.

## Clinical information

The patient is a 62-years-old male who was admitted to the hospital with episodes of abnormal mental behavior over the past 2 months. The patient experienced episodes of abnormal mental behavior, including two incidents where he wore a thick blanket while walking in the yard, struggled to find the car key, and frequently scraped the car against the wall. Additionally, he would sometimes leave home barefoot and had difficulty recalling the events afterward. He also reported dizziness, swelling, and limb weakness, along with slow walking. This morning, at around 5 a.m., the patient experienced urination in an unspecified location, which he could not recall. At approximately 6 a.m., he suddenly failed to recognize his family and left his room independently. Upon family intervention, he returned to normal. The patient, accompanied by family members, presented to our hospital for further diagnostic evaluation of “behavioral abnormalities.”

The patient had no history of hypertension, diabetes mellitus, or coronary heart disease. There was no history of drug allergy, trauma, surgery, blood transfusion, or blood donation. His personal history revealed no toxic exposure, with minimal smoking and alcohol consumption. On admission, neurological examination showed no localizing signs. The MMSE score was 21, and the MoCA score was 25. Laboratory results showed normal liver function, with glutamic transaminase at 41.1 U/L and glycosylated hemoglobin at 6.4%. Other tests, including blood lipids, renal function, cardiac function, electrolytes, blood routine, urine analysis, stool routine, coagulation profile, and thyroid function, were all normal. Infectious disease panel results showed Hepatitis B surface antigen quantification at 943.309 IU/ml, Hepatitis B core antibody > 10.0 IU/ml, Hepatitis B e antibody > 3.0 IU/ml, and Hepatitis B DNA quantification at 5970 IU/ml. Hepatitis C, syphilis, and HIV were negative. Tumor markers were within normal limits. Autoimmune liver disease tests were also negative.

**FIGURE 1:**
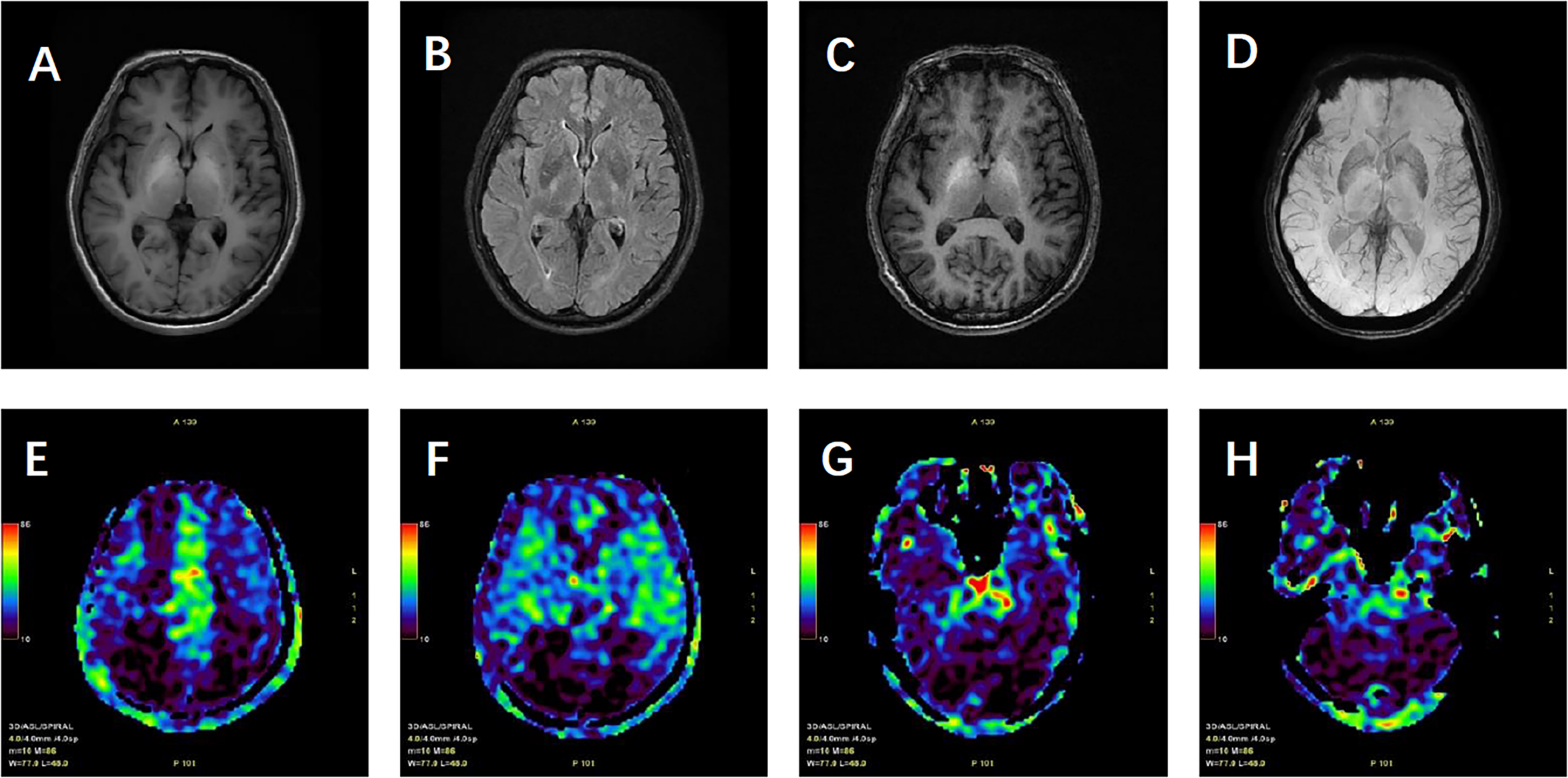
Patient’s cranial magnetic resonance imaging findings. **(A)** T1-weighted images (T1WI) showing high signal intensity with symmetrical distribution in the bilateral pallidum. **(B)** T2-weighted fluid-attenuated inversion recovery (T2 FLAIR) showing no abnormal high signal. **(C)** Contrast-enhanced T1WI (T1WI + C) demonstrating uniform enhancement of the bilateral pallidum. **(D)** Susceptibility-weighted imaging (SWI) with no significant abnormalities noted. **(E–H)** Arterial spin labeling (ASL) revealing hypoperfusion in the bilateral frontal, parietal, part of temporal, occipital lobes and cerebellum.

**FIGURE 2:**
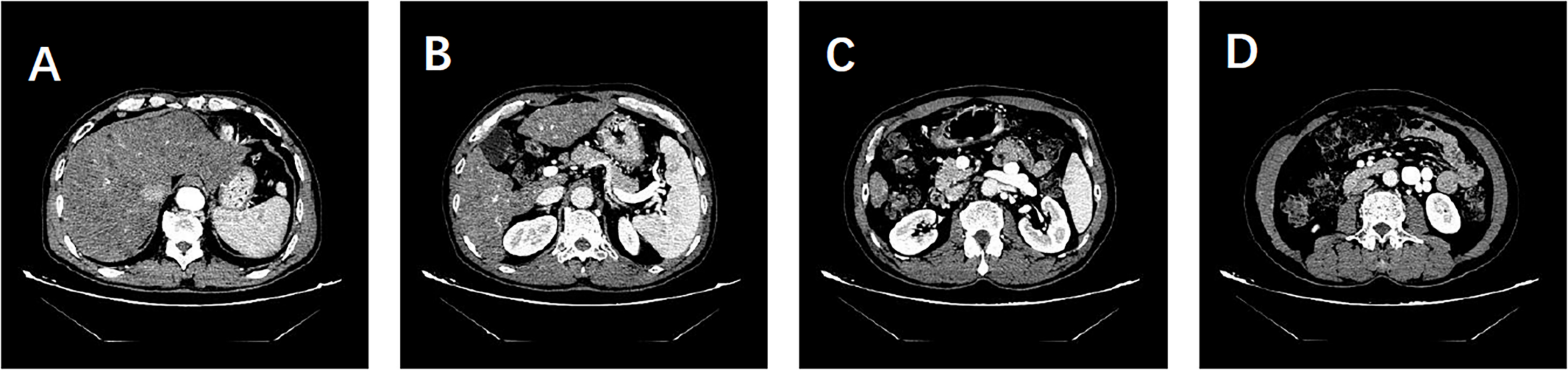
Patient’s hepatic artery CTA and portal vein CTV findings. **(A)** Hepatic artery CTA showing no abnormalities. **(B–D)** Portal vein CTV revealing dilatation of the splenic and renal veins and tortuous dilatation of the superior mesenteric vein. No evidence of portal shunt or splenorenal shunt is observed.

Cranial MRI revealed high signals in the T1WI sequence with symmetrical distribution in the bilateral pallidum, with no abnormal signals in the T2 Flair, DWI, or SWI sequences. Cranial ASL showed bilateral hypoperfusion in the frontal, parietal, and part of the temporal and occipital lobes. Enhanced cranial MRI demonstrated uniform enhancement of the bilateral pallidum (Figure 1). Hepatic artery CTA showed no abnormalities. Portal vein CTV revealed splenic and renal vein dilatation, and tortuous dilatation of the superior mesenteric vein (Figure 2). Electroencephalogram showed slowing of occipital rhythms with increased slow waves. The preliminary diagnosis upon admission was metabolic encephalopathy, and blood ammonia was elevated at 240 μmol/L.

**FIGURE 3:**
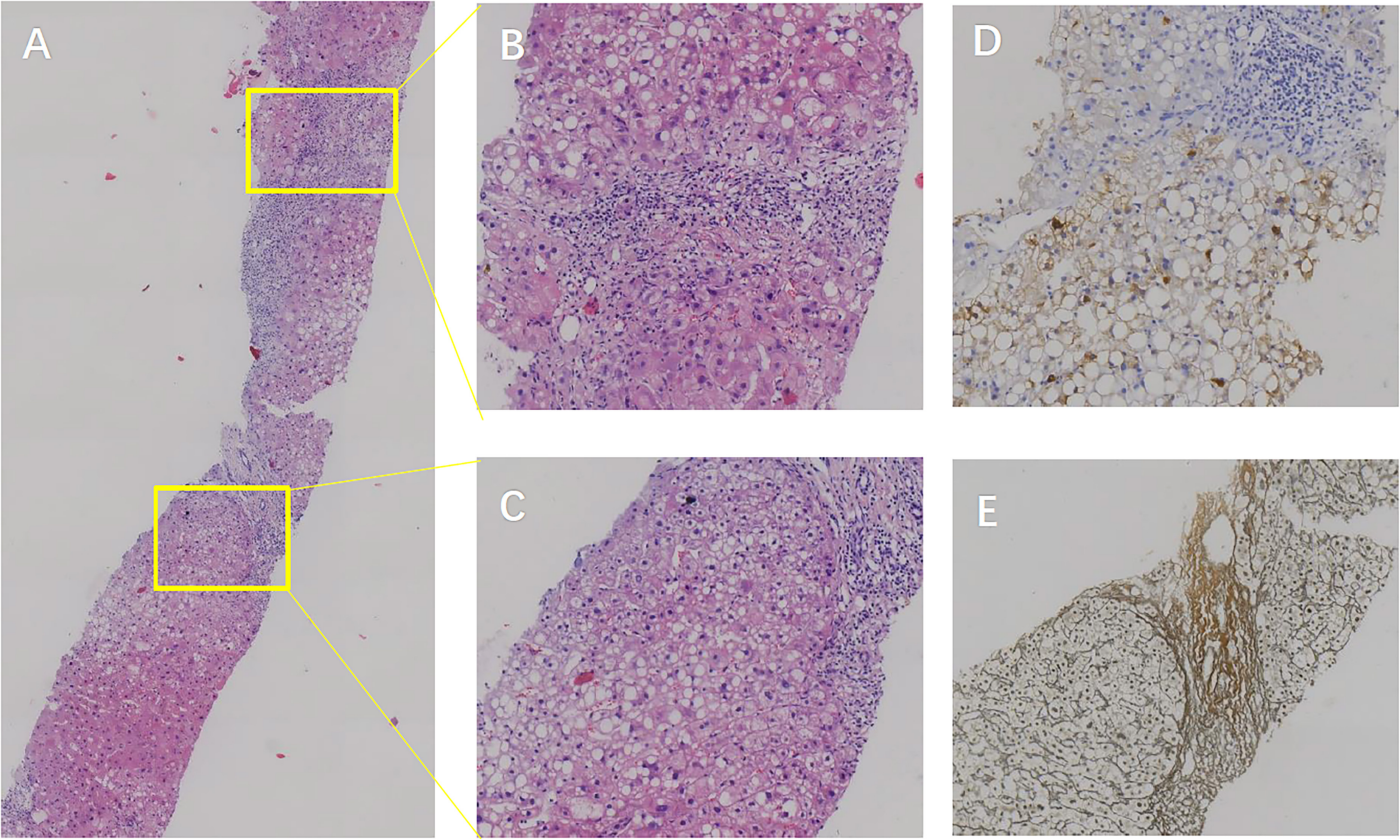
Patient’s liver biopsy findings. **(A)** Hematoxylin and eosin (H&E) staining at 5× magnification showing poorly arranged hepatic plates within lobules, hepatocyte cytoplasmic laxity, eosinophilic degeneration, and ballooning. Mallory vesicle formation and macrovesicular as well as vesicular steatosis are observed in lobule III. **(B,C)** H&E staining at 20× magnification revealing scattered moderate focal necrosis in hepatic lobules, with local bridging necrotic formation, mild central vein inflammation, active sinusoidal cellular reactions, lymphocytic infiltration in the sinusoids, and visible perisinusoidal fibrosis. The confluence area shows mild to moderate enlargement, lymphocytic and plasma cell infiltration, interstitial fibroplasia, fibrous septum formation, focal lobular structural disorders, and incomplete pseudolobule formation. **(D)** HBsAg staining at 20× magnification highlighting positive hepatitis B surface antigen expression. **(E)** Reticulocyte fiber staining at 20× magnification displaying positive reticulocyte fibers, indicative of fibrous activity.

Liver biopsy findings revealed poorly arranged liver plates, ballooning hepatocytes with Mallory’s hyaline bodies, and about 30% vesicular steatosis in hepatocytes in the III lobe, along with glycogen accumulation. The biopsy also revealed moderate focal necrosis and fibrosis in the hepatic lobules (Figure 3). According to the Chinese Guideline for the Diagnosis and Treatment of Liver Fibrosis and Cirrhosis (G/S system, comparable in principle to METAVIR), the pathological diagnosis was non-alcoholic steatohepatitis (NASH, F1G3S3-4, NAS score: 6) and moderate chronic hepatitis B (G2-3S3-4).

Considering the patient’s medical history, test results, and pathological findings, the final diagnosis was non-cirrhotic portal hypertension-associated hepatic encephalopathy due to non-alcoholic steatohepatitis combined with hepatitis B virus infection. The patient was treated with ornithine aspartate, rifaximin, lactulose, entecavir, and vitamin E. One week later, blood ammonia was reduced to 80 μmol/L, and symptoms were fully resolved. Upon discharge, the patient was advised to control his diet and continue oral lactulose medication. At the 6-months follow-up, he reported one episode of drug withdrawal.

## Discussion

Non-alcoholic steatohepatitis, HBV, and NCPH present significant challenges in clinical features and diagnosis. The symptoms of NASH are often atypical, including malaise and dyspepsia, with key pathological features such as hepatocellular steatosis, inflammation, necrosis, and hepatic fibrosis. Although liver biopsy remains the gold standard for diagnosing NASH, its clinical application is limited due to its invasive nature. Furthermore, HBV infection can lead to chronic liver diseases, such as hepatitis flare-ups and cirrhosis, which are closely linked to hepatocarcinogenesis. Challenges also exist in determining infection status and assessing liver lesions, including cryptic infections, viral mutations, and the difficulty in evaluating inflammation and fibrosis levels (2). NCPH primarily presents with symptoms of portal hypertension and hepatic encephalopathy, with vascular lesions being crucial for diagnosis. However, diagnostic criteria are difficult to define, and distinguishing it from hepatic fibrosis remains challenging. NCPH is characterized by symptoms of portal hypertension and hepatic encephalopathy. However, its diagnostic criteria are difficult to define, and it is challenging to differentiate it from cirrhosis. Additionally, the symptoms of hepatic encephalopathy are often atypical, and the available diagnostic markers are limited (1). Prior to hospital admission, the patient did not exhibit symptoms such as dyspepsia, weakness, nausea, or vomiting, presenting only with mental and behavioral abnormalities. It was only upon admission that HBV infection was detected. Moreover, ultrasound imaging did not indicate cirrhosis, which made the diagnosis challenging and led to an initial misdiagnosis of autoimmune encephalitis, as there were no obvious imaging clues to suggest hepatic encephalopathy. Later, based on imaging findings, elevated blood ammonia levels were detected. However, the insufficient evidence supporting hepatitis B-induced cirrhosis as the cause of classic hepatic encephalopathy led us to refine the hepatic arterial CTA and portal vein CTV to investigate hyperammonemia-associated encephalopathy and NCPH. Liver biopsy confirmed the diagnosis of NCPH, providing definitive etiological evidence for the patient’s condition.

There is a complex and intricate relationship between NASH, HBV, and NCPH. In terms of pathological development, each condition contributes to liver damage: NASH results from fat accumulation and metabolic disorders, leading to hepatocellular steatosis, inflammatory cell infiltration, and necrosis; HBV infection damages hepatocytes and induces inflammation and fibrosis through viral invasion and host immune response; NCPH impairs hepatic blood perfusion due to elevated portal vein pressure (4). These intertwined processes collectively contribute to the structural destruction and dysfunction of the liver, thereby increasing the risk of pathogenesis in each condition. For instance, Wang et al. demonstrated that liver fibrosis was more pronounced when NASH was coexistent with HBV infection (4). The pathohistological grading of the liver biopsy in our patient indicated advanced fibrosis (S3-4), consistent with the literature. Regarding metabolic disorders, both HBV infection and NASH can induce abnormalities in lipid and glucose metabolism. HBV infection can lead to altered lipid profiles, such as elevated triglycerides (TG) and reduced high-density lipoprotein cholesterol (HDL-C), whereas NASH is characterized by disordered lipid metabolism, manifesting as increased levels of triglycerides and free fatty acids in the blood. These metabolites, resulting from abnormal lipid metabolism, exert toxic effects on liver cells, activate inflammatory responses, and exacerbate liver inflammation (2, 4). Furthermore, both conditions are associated with insulin resistance, which leads to elevated blood glucose levels. The accumulation of lactic acid from abnormal glucose metabolism in the liver may lead to acidosis, further aggravating liver injury (1, 2). Our patient’s lipid levels were normal, potentially due to intermittent oral lipid-lowering medication and dietary management. In contrast, the patient’s Homeostasis Model Assessment of Insulin Resistance (HOMA-IR) was elevated, indicating insulin resistance, consistent with literature reports.

Imaging of hepatic encephalopathy (HE) plays a crucial role in elucidating its neurological mechanisms and in revealing associated structural and functional changes. Different forms of HE may show distinct imaging features (5). In cirrhotic HE, which represents a severe complication of chronic liver disease, prolonged inflammation and fibrosis are common findings, and a symmetrical high signal on T1-weighted images (T1WI) in the bilateral pallidum is considered a characteristic feature. These pallidal changes are thought to reflect manganese deposition and altered neurotransmitter metabolism, thereby linking the imaging pattern to the underlying metabolic encephalopathy. By contrast, non-cirrhotic portal hypertension (NCPH) is primarily related to elevated portal venous pressure with less prominent parenchymal injury, though it may involve marked vascular alterations such as portal vein stenosis and collateral vessel formation. The imaging characteristics of basal ganglia changes in NCPH remain less clearly defined and require further study (6). Patients with cirrhosis also tend to show more pronounced brain atrophy than those with NCPH, who usually present with only mild atrophic changes (7).

In our patient, the basal ganglia signal abnormalities on conventional MRI did not differ significantly from those typically observed in cirrhotic cases. This finding highlights that standard MRI sequences alone are insufficient for distinguishing cirrhosis-related HE from NCPH. To address this limitation, we emphasize the role of multimodal imaging, particularly two modalities that proved most critical for differentiation in our case: susceptibility-weighted imaging (SWI) and arterial spin labeling (ASL). SWI, highly sensitive to variations in magnetic susceptibility, allows visualization of hemorrhage, iron deposition, and subtle microvascular abnormalities, which are often more pronounced in NCPH (8). ASL, a non-invasive method for quantifying cerebral blood flow (CBF), provides functional information on perfusion changes. In our patient, ASL revealed reduced perfusion in the frontal, parietal, and occipital lobes, a novel observation consistent with the clinical presentation and potentially useful for ongoing monitoring of disease progression (9).

The treatment of hepatic encephalopathy, whether due to cirrhosis or non-cirrhotic portal hypertension, exhibits both similarities and differences. Overall, both treatments aim to enhance neurological function, reduce symptoms, and improve quality of life, while also preventing complications. Treatment strategies primarily focus on reducing ammonia production and absorption through medications such as lactulose and rifaximin, and managing portal shunts via vascular embolization and surgical interventions (10). However, the etiological and pathological bases of the two conditions differ significantly. Cirrhotic hepatic encephalopathy primarily originates from prolonged liver damage, hepatocellular failure, and portal shunts, characterized by pathological changes such as hepatic fibrosis. In contrast, non-cirrhotic portal hypertensive hepatic encephalopathy arises from abnormal shunts due to elevated portal pressure, typically without evident cirrhotic changes in the liver parenchyma. Regarding treatment focus and strategy, cirrhosis management emphasizes supporting liver function and managing complications, such as albumin supplementation and ascites control. This approach prioritizes individualization, tailoring the regimen to the disease’s severity and liver function status (11). Conversely, treating non-cirrhotic portal hypertension centers on managing vascular pathologies like thrombosis or stenosis, ensuring timely detection and intervention in significant shunts, and prioritizing recurrence prevention and enhanced monitoring (12). Therapeutic drug selection and timing for cirrhosis involve applying new ammonia-lowering agents and optimizing traditional medication use, with a focus on early intervention. For non-cirrhotic portal hypertension, treatment involves selecting targeted medications based on the vascular lesion characteristics, managing acute episodes promptly, and employing long-term strategies to prevent relapse. Additionally, the condition requires close monitoring during treatment, with treatment efficacy assessed through indicators including neurological function and blood ammonia levels.

In summary, NASH, HBV, and NCPH each present distinct challenges in clinical features and diagnosis. NASH is characterized by atypical symptoms, with liver biopsy being the gold standard for diagnosis, though its application is limited, and differential diagnosis is difficult. HBV infection status is challenging to assess, and liver lesions are difficult to evaluate. Furthermore, determining the diagnostic criteria for NCPH and differentiating it from liver cirrhosis remains complex. The relationship between these three diseases is intricate, as all contribute to liver damage and increase the risk of each other’s development. Both HBV and NASH contribute to metabolic disorders, causing abnormalities in lipid and glucose metabolism, and are associated with insulin resistance. Imaging plays a critical role in revealing the neurological mechanisms of hepatic encephalopathy. There are distinct differences in the imaging characteristics of hepatic encephalopathy caused by cirrhosis versus NCPH, and multimodal imaging is essential for differentiation. The treatment of hepatic encephalopathy due to cirrhosis and non-cirrhotic portal hypertension shares the same therapeutic goals; however, differences in etiology and pathology lead to variations in therapeutic focus, strategy, drug selection, and timing. The condition must be closely monitored throughout treatment.

## Data Availability

The datasets presented in this article are not readily available because of ethical and privacy restrictions. Requests to access the datasets should be directed to the corresponding authors.
